# Cell Wall Proteins Play Critical Roles in Plant Adaptation to Phosphorus Deficiency

**DOI:** 10.3390/ijms20215259

**Published:** 2019-10-23

**Authors:** Weiwei Wu, Shengnan Zhu, Qianqian Chen, Yan Lin, Jiang Tian, Cuiyue Liang

**Affiliations:** Root Biology Center, State Key Laboratory for Conservation and Utilization of Subtropical Agro-Bioresources, South China Agricultural University, Guangzhou 510642, China; weiweiwu2016@163.com (W.W.); shnzhu@163.com (S.Z.); qian2016200202@163.com (Q.C.); anlynlin@126.com (Y.L.); jtian@scau.edu.cn (J.T.)

**Keywords:** cell wall protein, phosphate starvation, Pi mobilization, roots

## Abstract

Phosphorus is one of the mineral nutrient elements essential for plant growth and development. Low phosphate (Pi) availability in soils adversely affects crop production. To cope with low P stress, remodeling of root morphology and architecture is generally observed in plants, which must be accompanied by root cell wall modifications. It has been documented that cell wall proteins (CWPs) play critical roles in shaping cell walls, transmitting signals, and protecting cells against environmental stresses. However, understanding of the functions of CWPs involved in plant adaptation to P deficiency remains fragmentary. The aim of this review was to summarize advances in identification and functional characterization of CWPs in responses to P deficiency, and to highlight the critical roles of CWPs in mediating root growth, P reutilization, and mobilization in plants.

## 1. Introduction

Phosphorus (P) is one of the mineral nutrients essential for plant development and growth, and serves as a major structural component and functions directly/indirectly in a set of biological and metabolic processes such as energy transmission, membrane and nucleotide synthesis, photosynthesis, and signal transduction [1]. However, due to its strong fixation and binding ability with organic matter and other minerals in soils, phosphate (Pi) bioavailability is often limiting, particularly in the highly-weathered acid soils in tropical and subtropical areas [2]. Therefore, low Pi availability becomes a major factor inhibiting crop production on arable lands, especially on acid soils [2,3]. To cope with low P stress on soils, plants have evolved a series of adaptive strategies to increase their Pi acquisition and utilization efficiency [4,5]. In plant roots, these multiple adaptive strategies to P deficiency mainly include: (i) using alternative metabolism pathways to reduce ATP consumption, (ii) stimulating root proliferation and enhancing symbiotic association with arbuscular mycorrhiza fungi to explore a more extensive volume of soil for Pi, (iii) enhancing root-mediated organic acid and purple acid phosphatase (PAP) exudation to remobilize sparingly-soluble P sources, and (iv) up-regulation of Pi transporters to enhance Pi uptake and translocation [1,4,5,6,7]. 

As part of the apoplast, which constitutes the extracellular space outside the plasma membrane, the cell wall is generally believed to play essential roles in cell division, enlargement, and differentiation. Meanwhile, the plant cell wall could also function as an important P pool for Pi reutilization when plants encounter P deficiency [8]. Therefore, many physiological and developmental changes of plant responses to Pi starvation are functionally related to cell wall modifications, and these modifications mainly determined by cell wall proteins (CWPs) [8,9,10,11,12]. For example, pectin content and the activities of pectin methylesterase (PMEs) in rice cell wall were found to be increased under low P conditions [11,12]. It was suggested that the carboxylate group (–COO–) in homogalacturonans can trap PO_4_^3−^ by forming a –COO–Fe–PO_4_^3−^ likage. The increasing amount of pectins in low P conditions might be able to provide more –COO–, which will bind Fe more tightly and subsequently release the trapped PO_4_^3−^ [8]. Therefore, the coordinated increase of PME activities under low P conditions has been suggested to provided more negative charges for the cell wall, which facilitates the release of cell-wall-bound Pi for reutilization [11,12,13].

With the aid of genetic, molecular, and proteomic analysis, numerous CWPs have been identified and functionally characterized [14,15,16,17,18,19,20]. In particular, great progress has been achieved in clarifying the function of CWPs in plant responses to various abiotic stresses [19,21,22,23,24]. Moreover, complex regulatory networks underlying CWP responses to stresses have been documented [19,25,26,27]. This review focuses on the characterization of Pi-starvation-responsive CWPs mediating plant adaptation to P deficiency. We briefly summarize the current methods for purification and identification of CWPs in plants. Subsequently, the role of CWPs in controlling plant adaptation to P deficiency has been discussed, especially for expansins, proline-rich proteins (PRPs), oxidoreductases, and purple acid phosphatases.

## 2. Extraction and Identification of CWPs in Responses to Pi Starvation 

Proteins localized in cell walls are ubiquitous in plants. Early studies characterized CWPs as proteins unusually rich in one or two amino acids, containing highly repetitive sequence domains, and glycosylated to varying degrees [14]. The most common types of CWPs include hydroxyproline-rich glycoproteins (HRGPs) or extensins, the arabinogalactan proteins (AGPs), the glycine-rich proteins (GRPs), the proline-rich proteins (PRPs), and chimeric proteins that contain extensin-like domains [14,28]. With assistances of the development of molecular, genetic, and proteomic technologies, a large number of other CWPs have been identified and the classical CWPs are understood to harbor several properties, including containing an N-terminal cleavage signal peptide, but lacking the ER-retention C-terminal motif KDEL/HDEL and transmembrane domains [18,20,29,30,31,32]. 

According to their binding ability to cell walls, plant CWPs can be classified into three categories: labile proteins, weakly bound proteins, and strongly bound proteins [18,30]. Due to these properties, different extraction approaches have been separately developed and widely applied to isolate diverse CWPs from plants [18,24,30,33,34,35,36]. Feiz et al. (2006) developed an efficient method for extracting weakly bound CWPs, in which several sequential steps are conducted, including plant sample homogenization in low ionic strength acid buffer, purification of cell walls through increasing density cushions, extensive washing of cell walls with a low ionic strength acid buffer to remove cytosolic protein contamination, and using CaCl_2_ and high concentration of LiCl to extract CWPs instead of detergents. Compared to other available methods of CWP extraction, this method increases the proportion of the potential CWPs while decreasing the intracellular protein contamination [33]. This method has been successfully and widely used to extract CPWs from a broad range of plants, such as potato (*Solanum tuberosum*), flax (*Linum usitatissimum*), and soybean [37,38,39]. However, it could not be avoided losing about 15% CWPs during cell wall purification and protein extraction because of the complexity of cell wall components and the diversity of CWP properties in plants [37]. Therefore, more efficient methods for CWP purification and isolation are yet to be designed.

Compared to CWP extraction, the approaches for separation, identification, and quantification of CWPs are better developed [40,41,42]. With the aid of various proteomics techniques, CWPs have been widely identified in different organs of many plant species, including Medicago (*Medicago truncatula*) [17,18,30,36,43,44,45,46], maize (*Zea mays*) [47], chickpea (*Cicer arietinum*) [48], rice (*Oryza sativa*) [34,49,50,51,52], potato [53], flax [54], and sugarcane (*Saccharum officinarum*) cell suspension cultures [55]. These identified CWPs can be functionally classified as proteases, structural proteins, proteins functioning in carbohydrate metabolism, proteins acting as oxidoreductases, proteins associated with lipid metabolism, proteins related to signaling transduction, proteins with predicted interaction domains, miscellaneous proteins, and unknown function proteins [18]. The diverse biological functions of CWPs indicate sophisticated modulations of the cell wall during plant development.

In addition, proteomics techniques have been widely used to identify CWPs differentially accumulated in responses to abiotic stresses in plants. For instance, Zhu et al (2007) [56] used 2D electrophoresis to separate the CWPs from water-deficit maize primary roots, and subsequently applied time-of-flight mass spectrometry to identify the differentially accumulated proteins. The results showed that a total of 152 water-deficit-responsive CWPs were identified, which were functionally categorized into proteins related to reactive oxygen species (ROS) metabolism, proteins involved in defense repsonse, hydrolases, proteins associated with carbohydrate metabolism, and other/unknown proteins [56]. Similar proteomic studies were also conducted to quantify and characterize CWPs in plant responses to a set of stresses, such as dehydration stress in maize [56], chickpea [57], and rice [58], flooding stress in both wheat (*Triticum aestivum*) [22] and soybean (*Glycine max*) [59], and hydrogen peroxide stress in rice [60]. The results indicate that stress-responsive CWPs mediate multiple processes, and thus regulate the responses of cell elongation and expansion under abiotic stress conditions.

Despite the cumulative studies on the identification of differentially accumulated CWPs in response to abiotic stresses, few studies have been conducted to globally identify CWPs in responses to nutrient deficiencies using proteomic approaches, particularly for P deficiency. Recently, a pioneer study was conducted to identify Pi-starvation-responsive and weakly bound CWPs in soybean roots through proteomic analysis [39]. The CWPs were extracted from soybean roots following the methods developed by Feiz et al. [33] and were subsequently analyzed by isobaric tag in relative and absolute quantitation (iTRAQ) proteomics techniques. A total of 71 differentially accumulated proteins were identified, among which, 53 proteins (74.6%) were predicated to be secreted and were considered cell-wall-associated proteins by bioinformatic analysis. These proteins were predicted to be involved in carbohydrate metabolism, oxido-reduction, protein modification and turnover, signal transduction, miscellaneous functions, and unknown functions [39]. The results strongly suggest that CWPs may regulate the complex responses in soybean roots under low P conditions. However, except for the purple acid phosphatase1 like (PAP1-like) protein, which was subsequently characterized, the functions of all other CWPs in soybean adaptation to low P stress remain unclear [39].

## 3. CWPs Related to Root Growth in Responses to Pi Starvation

Changes of root morphology and architecture have been widely observed in plants in response to Pi starvation, which extensively increases root proliferation for soil exploration [4,61]. The modulation of root morphology and architecture is highly related to root cell wall synthesis and remodeling, which mainly relies on coordinated regulation of CWPs’ enzymatic activities. However, differences of root growth in response to Pi starvation among various plant species could lead to more complex regulatory networks. For example, in P-deficient Arabidopsis, growth of primary root is arrested, while growth of lateral roots and root hairs is promoted [62]. For other plant species, such as rice and soybean, both the primary root and lateral (adventitious) root length are increased by short-term Pi starvation [39,63]. Moreover, white lupine (*Lupinus albus*) develops large numbers of cluster roots under Pi starvation conditions [64]. Thus, different responses of root morphology in various plant species should be also taken into consideration when functions of CWPs are characterized in plant acclimatization to low P stress. 

So far, the functions of most CWPs in mediating plant root growth in responses to Pi starvation remain largely unknown, except for a small set of CWPs including expansins, PRPs, and some oxidoreductases [65,66,67,68,69].

### 3.1. Expansin

Expansins have been widely identified in plant cell walls, and are believed to loosen the cell wall by breaking the hydrogen bonds between hemicellulose and cellulose microfibrils in a pH-dependent manner, thus enabling turgor-driven cell expansion [70,71]. Since increases in cell size are considered to be essential for plant root growth, it is generally assumed that expansins play a critical role in mediating root growth and development. In plants, expansins are classified into four sub-families, including α-expansin or expansin A (EXPA), β-expansin or expansin B (EXPB), expansin-like A (EXPLA), and expansin-like B (EXPLB) [71,72]. 

Since the first expansin protein was identified in cucumber (*Cucumis sativus*) hypocotyls [73], diverse biological functions of expansin members have been widely found to be associated with environmental stresses in plants [71]. However, the roles of expansins in plant adaptation to P deficiency has attracted little attention. A β-expansin gene from soybean, *GmEXPB2*, was the first one found to regulate root growth in responses to Pi starvation [65]. It was reported that *GmEXPB2* was highly induced by Pi starvation. Transgenic Arabidopsis with *GmEXPB2* overexpression exhibited increased root cell division and elongation, which subsequently enhanced plant growth and Pi uptake [65]. A follow-up report provided supporting evidence that transgenic soybean with *GmEXPB2* overexpression also had higher plant dry weight and P content compared to wild-type plants when gown in calcareous soils [66]. Similar results were reported in wheat coleoptiles: that multiple expansins (five α- and nine β-expansin genes) were up- or down-regulated in responses to Pi application [74]. Overexpression of one expansin member, *TaEXPB23*, significantly increased the number of lateral roots in transgenic tobacco (*Nicotiana tabacum*) under both high and low P conditions [74]. These results strongly suggest the involvement of expansins in mediating root growth in responses to P deficiency.

Besides enhancing tap or lateral root elongation, root hair growth is also regulated by expansins. In soybean, GmEXPB2 was suggested to play roles in the process of soybean root hair development basing on the observation that overexpression of *GmEXPB2* dramatically increased root hair density and size of the root hair zone [75]. Similar functions were also reported for a group of expansins in other plants [71]. For example, AtEXPA7 in Arabidopsis and OsEXPA17 in rice have been documented to be required for root hair elongation [76,77], while HvEXPB1 in barley has been demonstrated to be root-hair-specific and to play roles in root hair formation [78,79]. Since increased root hair density and length is considered to improve Pi uptake efficiency in plants [80,81], root hair growth and development regulated by expansins presents a good example in which CWPs play a critical role in mediating root growth in responses to low P stress. 

### 3.2. Proline-Rich Proteins

PRPs is a protein family with Pro- and Hyp-rich amino acid sequences. Following the identification of the first PRP in carrot (*Daucus carrota*) storage roots [82,83], a number of PRPs have been characterized in many plant species such as soybean [84,85,86,87,88,89], bean (*Phaseolus vulgaris*) [90], Medicago [91], pea (*Pisum sativum*) [86,92], maize [93], tomato (*Lycopersicon esculentum*) [94,95,96], and Arabidopsis [97,98,99]. PRPs have multiple functions, and are assumed to participate in plant development and growth, nodule formation, pathogen infection, and abiotic stress and wounding responses [83,86,90,91,92,100,101,102,103]. 

Although the roles of PRPs in response to biotic and abiotic stresses have been well documented, little information is available on their functions in plant adaptation to P deficiency. Recently, a SRPP protein, which is partially homologous to PRPs but lacks the proline-rich domain in its N-terminal region, was reported to function in Arabidopsis responses to Pi starvation [69]. It was found that SRPP is localized in cell walls, and its transcripts are specifically accumulated in both root hairs and fruits. Furthermore, loss function of *SRPP* results in shorter root hair length and reduced root hair viability under Pi starvation conditions, strongly suggesting that SRPP is involved in the regulation of root hair growth and development under low P conditions [69]. Although functions of other PRPs in plant adaptation to Pi starvation remain largely unknown, it could be hypothesized that they might play a role in plants’ adaptation to Pi starvation, because transcripts of other *PRP* or *PRP*-like genes were found to be up-regulated by Pi starvation through transcriptomic analysis in plants such as Arabidopsis [104]. 

### 3.3. Oxidoreductases 

A number of CWPs associated with oxido-reduction have been identified and functionally characterized in plant responses to Pi starvation [39,67]. A typical example is the cell-wall-localized ferroxidase LPR1 (Low Phosphate Root1), which has been well documented to play a critical role in cell-wall-mediated primary root responses to Pi starvation in Arabidopsis [67]. During Pi limitation, LPR1 modulates Fe deposition in cell walls of the root apical meristem and elongation zone, and thus leads to an oxidative environment for enhancing reactive oxygen species (ROS) generation. Meanwhile, increased accumulation of ROS in the apoplast subsequently induces callose deposition in cell walls and finally impairs SHORT ROOT protein movement and the maintenance of stem cell niche in root apical meristem [67]. On the other hand, ROS may also enhance the activity of class III peroxidases in the cell wall during Pi starvation, which could use H_2_O_2_ as a co-substrate and stiffen cell walls by catalyzing the cross-link between cell wall components [68,105]. However, since lateral root responses to Pi starvation are quite different from those of the primary roots, it still remains unclear whether cell wall modifications mediated by the LPR1-modulated ROS pathway are applicable to lateral root development under low P conditions.

Meanwhile, with the aid of transcriptomic and proteomics analysis, Pi-starvation-responsive peroxidases have been observed in plants [39,106,107]. For example, proteomics analysis of CWPs in soybean roots revealed that abundance of five peroxidase isoforms was either increased or decreased by Pi starvation, indicating a possible role of these peroxidases in soybean cell wall modification in response to low P stress [39]. Similar results were also obtained in cotton (*Gossypium hirsutum*): that 180 peroxidases were predicted to be secreted into the apoplast [107]. Among them, the expression patterns of 18 peroxidase genes were significantly influenced by P deficiency [107]. The results indicate complex regulation and diverse functions of peroxidases in responses to Pi starvation, suggesting that it is worth to investigating their functions in plant cell wall modification under low P conditions. 

In addition, a cell-wall-targeted berberine bridge enzyme (BBE)-like protein was demonstrated to be regulated by Pi starvation in soybean roots [39]. It was revealed that protein abundance of the BBE-like protein was increased by about 6-fold under low P conditions, strongly suggesting that BBE-like protein might participate in root responses to Pi starvation [39]. The BBE protein in plants was first functionally characterized in poppy (*Eschscholzia californica*) in which it oxidates the N-methyl group of (S)-reticuline, yielding (S)-scoulerine [108]. In recent years, numbers of genes encoding BBE-like enzymes have been identified in plants, but functions of BBE-like proteins in plants are largely unexplored. In Arabidopsis, a total of 28 *BBE*-like genes have been identified [109]. The transcripts of *AtBBE*-like genes were found by microarray assay at different root developmental stages, such as lateral root initiation, root elongation and maturation [109,110]. Moreover, four members of this group, including AT1G01980, AT4G20840, AT1G11770, and AT4G20830, exhibit the ability to oxidize oligogalacturonides, thus enhancing resistance to fungi [111]. Additionally, oligogalacturonides have been demonstrated to play a role in root elongation by inducing the oxidative burst in alfalfa (*Medicago sativa*), suggesting that BBE-like proteins might have the potential role of controlling root growth by mediated the dynamic equilibrium of oligogalacturonides in cell walls [112]. However, the functions of BBE-like proteins in plant root development in responses to Pi starvation remain enigmatic.

## 4. Purple Acid Phosphatase Functions in Cell Wall Synthesis and Pi Mobilization

Another plant adaptation strategy to low P stress is to increase the accumulation of extracellular acid phosphatase (APase), which facilitates the scavenging of Pi from organophosphate compounds in apoplasts [4,113]. 

PAPs belong to the APase family, which exhibits distinct biochemical properties, such as purple color in aqueous solution, activity insensitive to inhibition by L-tartrate, and a bimetallic active site [113]. The plant PAP family is composed of a large group of members and displays non-specific APase activities, which catalyze the hydrolysis of Pi from a wide range of organophosphates, with optimum reaction pH below 7.0. Although diverse functions of plant PAPs have been observed in plants, most plant PAPs are suggested to play a critical role in P scavenging and recycling in plants, especially for extracellular PAPs [113,114]. 

A group of cell-wall-targeted PAPs has been well characterized in plants, including Arabidopsis, soybean, and tobacco [113]. In Arabidopsis, 14 PAPs have been identified in cell wall proteomes (WallProtDB, http://www.polebio.lrsv.ups-tlse.fr/WallProtDB/). Among them, four PAPs, including AtPAP10, AtPAP12, AtPAP25, and AtPAP26, have been functionally characterized in Arabidopsis responses to Pi starvation [115,116,117,118,119]; AtPAP12 and AtPAP26 contribute to over 70% of the total APase activity secreted by roots or targeted to shoot cell walls of Arabidopsis under low P conditions [115,117]. Furthermore, an *atpap12/atpap26* double mutant exhibited impaired growth when organic P (e.g., glycerol-3-phospahte and herring sperm DNA) was applied as the sole P source, strongly suggesting that cell-wall-associated AtPAP12 and/or AtPAP26 participates in recycling Pi from endogenous phosphomonoesters that leak into cell walls [118,120]. Similarly, AtPAP10 is predominantly localized to the surface of root epidermal cells and functions in the acclimation of Arabidopsis to Pi deprivation through activating organic P in solid MS medium [116,117].

Recently, an iTRAQ proteomics analysis was conducted to identify CWPs with differential accumulations in soybean roots in response to Pi starvation [39]. Among differential CWPs, a cell wall-localized PAP, GmPAP1-like, was identified to be up-regulated by Pi starvation. Furthermore, *GmPAP1-like* overexpression resulted in significant increases of both relative growth and P content in soybean hairy roots when deoxy-ribonucleotide triphosphate (dNTP) was supplied as the sole external P source, strongly suggesting that cell-wall-targeted GmPAP1-like participates in extracellular organic-P utilization in plants [39]. 

However, unlike GmPAP1-like, AtPAP12, and AtPAP26, a cell-wall-targeted PAP, AtPAP25, exhibited high phosphatase activity against several phosphoproteins and phosphoamino acids in vitro. Moreover, mutation of *AtPAP25* led to a reduced level of transcripts encoding *At4*, *AtPPCK1*, *AtRNS1*, *AtPAP12*, and *AtPAP17*, which were involved in Pi starvation responses. Therefore, it was suggested that AtPAP25 might function as a phosphoprotein phosphatase and play important signaling roles during Pi deprivation [119]. Similarly, a cell wall localized PAP, NtPAP12, was purified in tobacco [121]. Furthermore, it was found that NtPAP12 could dephosphorylate several cell-wall-bound enzymes, such as α-xylosidase and β-glucosidase, in vitro, suggesting that NtPAP12 might be involved in the regulation of cell wall biosynthesis [122]. 

Therefore, it seems that functional dissection of cell-wall-targeted PAPs will be a complex task because functions vary among PAPs owing to their respective protein structures, accumulation patterns, and a wide variety of potential substrates. Furthermore, considering that several substrates of plant PAPs have been suggested to be signaling molecules, such as ATP, it is reasonable to hypothesize that PAP could influence signaling molecule metabolism, and thus participate in the complex signaling network in plants. Therefore, identification and characterization of cell-wall-targeted PAPs will be helpful in elucidating the functions of PAP in plant acclimation to P deficiency [113]. 

## 5. Conclusions and Perspectives

Great progress has been made in revealing the important roles of CWPs in the modification of cell wall properties, which is crucial for the meticulous modulation of plant roots in response to P deficiency. For example, LPR1-mediated callose deposition results in the inhibition of primary root growth under Pi-starvation conditions in Arabidopsis [67]. However, the primary root growth responses of other plant species are quite different from those of Arabidopsis [39,63]; whether the same regulatory pathway also exists in other plant species remains for further studies to determine. Moreover, the plant cell wall is a complex matrix, mainly consisting of cellulose microfibrills and other wall components, such as pectins, hemicelluloses, callose, and lignin, depending on the cell wall type [14,27,123]. When plants were subjected to Pi starvation, the cell wall components were changed, thus changing the cell wall’s physical properties, and subsequently influencing cell expansion [8,68]. Therefore, to understand the roles of CWPs in root modulation under low P conditions, future studies are expected to investigate how CWPs concomitantly modulate the biochemistry and physiological properties of the cell wall components.

In order to elucidate the functions of CWPs in cell wall adaptation to Pi starvation, identification of CWPs with differential accumulation is critical. However, few proteomic studies have been conducted to identify CWPs in response to Pi starvation, thus limiting the understanding of the functions of CWPs in plant adaptation to Pi starvation. Meanwhile, since covalently bound proteins are poorly extracted, the full coverage of plant cell wall proteomes remains challenging [37]. A better extraction protocol remains to be developed to increase this coverage.

Taken together, the studies referenced here have demonstrated that CWPs play important roles in plant responses to Pi starvation. It can be anticipated that increased knowledge in this field will provide important theoretical bases for engineering crops with increased P efficiency.
